# Targeting Atherosclerosis: Cholesterol-Lowering Therapies with a New Immunometabolic Dress for an Old Disease

**DOI:** 10.3390/jcm14248777

**Published:** 2025-12-11

**Authors:** Josep Julve, Ricardo Rodriguez-Calvo, Bertrand Perret, Laurent O. Martinez, Didac Mauricio

**Affiliations:** 1 Research Group in Endocrinology, Diabetes and Nutrition, Institut de Recerca Sant Pau, 08041 Barcelona, Spain; 2 Spanish Biomedical Research Center in Diabetes and Associated Metabolic Diseases (CIBERDEM), Instituto de Salud Carlos III, 28029 Madrid, Spain; ricardo.rodriguez@ciberdem.org; 3 Research Unit on Lipids and Atherosclerosis, University Rovira i Virgili, 43201 Reus, Spain; 4 Vascular Medicine and Metabolism Unit, “Sant Joan de Reus” University Hospital, 43204 Reus, Spain; 5 Pere Virgili Health Research Institute (IISPV), 43007 Tarragona, Spain; 6 LiMitAging, Institute of Metabolic and Cardiovascular Diseases, I2MC, Université de Toulouse, Institut National de la Santé et de la Recherche Médicale (INSERM), UMR1297, 31000 Toulouse, France; bertrand.perret3@free.fr (B.P.); laurent.martinez@inserm.fr (L.O.M.); 7 Institut Hospitalo-Universitaire (IHU) HealthAge, 31000 Toulouse, France; 8 Department of Endocrinology and Nutrition, Hospital de la Santa Creu i Sant Pau, 08041 Barcelona, Spain; 9 Faculty of Medicine, University of Vic—Central University of Catalonia, 08500 Vic, Spain

**Keywords:** lipids, lipoprotein metabolism, cardiovascular disease, PCSK9, statins, inflammation, diabetes, immune cells, glycoproteins, plaque, clinical studies

## Abstract

Atherosclerotic cardiovascular disease (ASCVD) persists as the foremost cause of global morbidity and mortality. Central to its pathogenesis, atherosclerosis emerges as a chronic inflammatory disorder fueled by the intricate interplay between dysregulated lipid metabolism and immune cell activation. Recent insights reveal that inflammatory cues within atherosclerotic plaques or ischemic tissues orchestrate metabolic reprogramming in immune cells, thereby modulating disease trajectories. While cholesterol-lowering agents such as statins and proprotein convertase subtilisin/kexin 9 (PCSK9) inhibitors have long been recognized for their lipid-modulating properties, accumulating evidence now underscores their pleiotropic anti-inflammatory effects mediated through immune cell modulation. For instance, recent clinical observations reveal that PCSK9 inhibitors not only substantially reduce low-density lipoprotein cholesterol (LDL-C) and triglycerides but also appear to reduce advanced glycoprotein signals, emerging composite biomarkers of systemic inflammation. This highlights a novel and more nuanced dimension of inflammation modulation by PCSK9 inhibitors, although current evidence remains limited and requires further confirmation. Moreover, this dual immune-metabolic influence reshapes our understanding of therapeutic mechanisms and calls for a reassessment of treatment paradigms in ASCVD management. Here, we present a synthesis of current findings that emphasize how both established and novel therapies transcend lipid-lowering to exert profound immunomodulatory actions, offering promising avenues to attenuate cardiovascular disease progression through integrated metabolic and inflammatory control.

## 1. Introduction

Atherosclerotic cardiovascular disease (ASCVD) remains the leading cause of morbidity and mortality worldwide. According to the Global Burden of Disease Study 2019 [1], cardiovascular diseases account for about one-third of all global deaths, with ischemic heart disease and ischemic stroke being the main contributors. In this context, atherosclerosis arises from a complex interplay between lipid accumulation and immune activation [2]. Deposition and modification of low-density lipoprotein (LDL) cholesterol (LDL-C) and lipoprotein remnant within the arterial intima initiate monocyte recruitment and foam cell formation, thereby promoting plaque development [3,4]. In macrophages, dysregulated cholesterol metabolism induces metabolic stress, inflammasome activation, enhances cytokine secretion, and sustains vascular inflammation [5,6]. This close connection between cholesterol metabolism and immune cell function defines the emerging field of immunometabolism, which offers novel therapeutic opportunities [7,8].

Within the immune-metabolic paradigm of atherosclerosis, recent studies have highlighted the potential diagnostic/prognostic value of glycated proteins GlycA and Glyc B (Table 1). These glycoproteins have been proposed as low-grade inflammation biomarkers that may be more informative than single molecules, as they integrate both the aggregation state and the protein levels of several abundant acute-phase proteins in serum [9]. Advanced proton nuclear magnetic resonance (^1^H-NMR) analyses in patients with type 1 diabetes (T1D) [10], together with large population studies of immunoglobulin G (IgG) glycosylation [11] have shown that GlycA, GlycB and specific IgG glycan traits are associated with carotid and femoral plaque burden independently of LDL-C and other conventional risk factors. These findings suggest that altered glycosylation patterns capture immune-metabolic dysregulation and inflammatory pathways in the vessel wall, providing information beyond traditional lipid measures. Conversely, some glycated protein markers appear more closely linked to lipid-driven atherogenesis. For example, glycated apolipoprotein (Apo)B correlates strongly with carotid intima-media thickness and plaque presence in type 2 diabetes [12], but this effect appears largely mediated by concomitant dyslipidemia. Similarly, data from the Multi-Ethnic Study of Atherosclerosis (MESA) cohort showed that GlycA is associated with subclinical peripheral arterial disease and prevalent carotid plaques, although these associations were substantially attenuated after adjustment for LDL and other lipid parameters [13].

**Figure 1:**
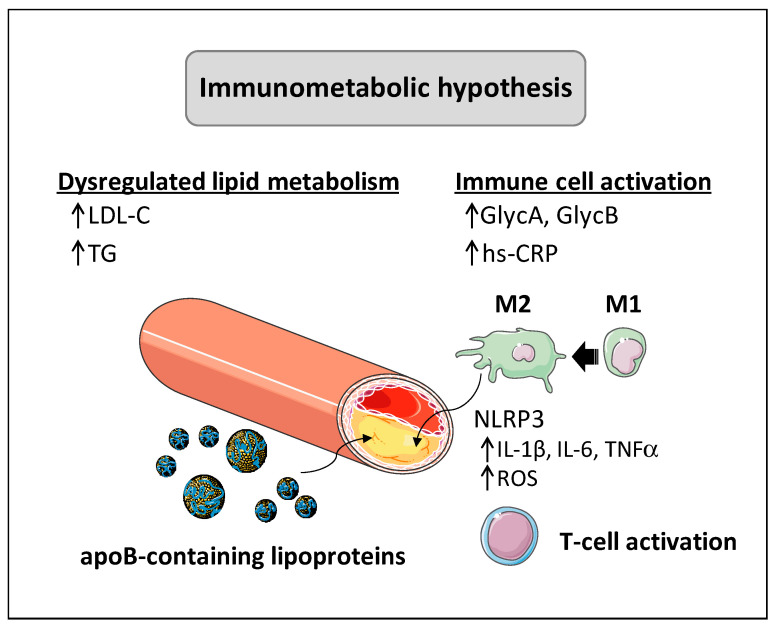
Immune-metabolic hypothesis. Both dysregulated lipid metabolism and immune cell activation intersect to drive atherosclerosis progression. ApoB-containing lipoproteins are captured and modified by activated macrophages (M2) in the subendothelial space of arteries, giving rise to foam cell-derived atherosclerotic plaque. This process promotes activation of the NLRP3-containing inflammasome and triggers the release of pro-inflammatory mediators (IL-1β, IL-6, TNFα) and reactive oxygen species, thereby promoting T cell activation and the inflammatory state characteristic of atherosclerotic disease. Concomitantly, the atherosclerotic process is related to increased levels of hs-CRP and glycated proteins such as GlycA and Glyc B, composite biomarkers reflecting low-grade inflammation better than single molecules. When shown, an upward arrow (↑) represents upregulation. Abbreviations: GlycA, glycoprotein A; GlycB, glycoprotein B; hs-CRP: high-sensitivity C-reactive protein; IL-1β, interleukin 1β; IL-6, interleukin 6; LDL-C, low-density lipoprotein-cholesterol; NLRP3, nucleotide-binding oligomerization domain (NOD)-, leucine-rich repeat (LRR)- and pyrin domain-containing protein 3; ROS, reactive oxygen species; TG, triglycerides; TNF-α, tumor necrosis factor α. This figure was created from its Servier items.

Together, these observations may support a dual scenario: certain glycated and glycosylated protein markers behave as lipid-independent indicators of arterial inflammation and remodeling, whereas others appear to amplify lipid-driven atherosclerosis. This duality underscores the potential of these novel molecules as integrative biomarkers of immune-metabolic dysregulation in atherosclerosis (Figure 1).

Cholesterol-lowering drugs, i.e., statins and proprotein convertase subtilisin/kexin type 9 (PCSK9) inhibitors, effectively reduce circulating LDL-C levels, although they act via different mechanisms. While statins inhibit de novo cholesterol synthesis by targeting 3-hydroxy-3-methylglutaryl-coenzyme A (HMG-CoA) reductase and also promote LDL receptor (LDLR) expression, PCSK9 inhibitors block the action of PCSK9, preventing LDLR degradation. This allows more receptors to remain on the hepatocyte membrane surface, thereby enhancing LDL-C removal from the circulation. Beyond their cholesterol-lowering action, both statins and PCSK9 inhibitors have increasingly been recognized for their pleiotropic actions, including modulation of vascular inflammation and stabilization of atherosclerotic plaques. Recent studies have highlighted that these therapies not only reduce LDL-C levels but also exert anti-inflammatory and anti-atherogenic effects, contributing significantly to attenuation of atherosclerotic progression [10,14,15,16,17,18,19,20,21,22,23]. Notably, statin therapy has been shown to influence glycoprotein biomarkers of systemic inflammation, such as GlycA and GlycB, measured by ^1^H-NMR spectroscopy. For instance, statin therapy has been associated with a pro-inflammatory pattern in the IgG glycome, indicating potential modulation of glycosylation and inflammatory processes [14]. GlycA and GlycB height/width ratios have been linked to carotid atherosclerosis, with associations influenced by background statin therapy [10], while statins led to a modest reduction in GlycA levels, suggesting limited but measurable effects on systemic inflammation [24]. Similarly, a study on PCSK9 inhibitors demonstrated a reduction in pro-inflammatory protein expression within atherosclerotic plaques, suggesting beneficial remodeling of the vascular inflammatory milieu independent of LDL-C lowering [16]. Collectively, these findings underscore that both statins and PCSK9 inhibitors contribute to cardiovascular protection not only through lipid lowering but also via direct modulation of immune-metabolic pathways. However, whether these anti-inflammatory effects are truly pleiotropic or largely a consequence of LDL-C reduction remains unclear. As highlighted in Table 1, some studies suggest that these therapies may exert direct anti-inflammatory effects, although the underlying mechanisms remain largely unknown and warrant further investigation.

**Figure 2:**
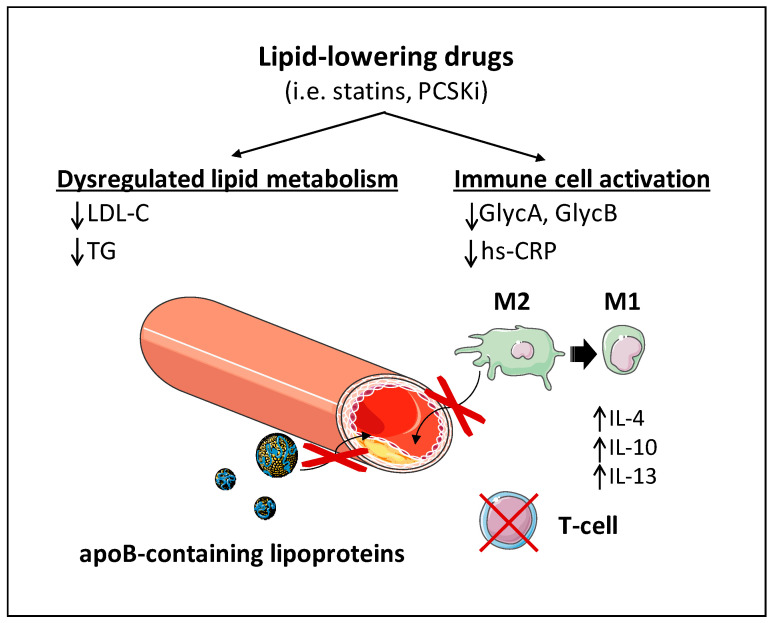
Lipid-lowering therapies’ impact on both dysregulated lipid metabolism and immune cell activation. Apart from their well-known hypolipidemic role, lipid-lowering drugs such as statins and PCSK inhibitors directly regulate immune cell activation. Lipid-lowering drugs promote an anti-inflammatory macrophage (M1) phenotype, thereby reducing its infiltration in the subendothelial space, the apoB-containing lipoproteins modification, and T-Cell activation. When shown, an upward arrow (↑) represents upregulation, and a downward arrow (↓) represents downregulation. Abbreviations: GlycA, glycoprotein A; GlycB, glycoprotein B; hs-CRP: high-sensitivity C-reactive protein; IL-4, interleukin 4; IL-10, interleukin 10; IL-13, interleukin 13; LDL-C, low-density lipoprotein-cholesterol; PCSKi, proprotein convertase subtilisin/kexin 9 inhibitors; TG, triglycerides. This figure was created from its Servier items.

It is important to note that the intended scope of this Perspective is not to provide a comprehensive overview of lipid-lowering strategies or their clinical combinations for cardiovascular risk reduction, as these topics have been extensively reviewed elsewhere. Instead, this Perspective reviews the evidence of the anti-inflammatory effects of lipid-lowering therapies, emphasizing that agents widely used in clinical practice not only reduce cholesterol levels but may also exert direct anti-inflammatory actions (Figure 2). We further propose hypotheses for future research using a holistic immune-metabolic perspective in atherosclerosis.

## 2. Towards the Immune-Metabolic Hypothesis of Atherosclerosis

Atherosclerosis is currently recognized as a chronic inflammatory disease intimately linked to dysregulated lipid metabolism. According to the classical view of plaque progression, persistent inflammatory stimuli, driven by hypercholesterolemia and endothelial dysfunction, promote the recruitment and activation of immune cells such as macrophages and T lymphocytes in the subendothelial space [5,6]. These activated cells release pro-inflammatory cytokines and reactive oxygen species, further exacerbating vascular injury and contributing to plaque instability [25]. This chronic inflammatory environment not only accelerates lesion development but also predisposes to plaque rupture and thrombosis, which are the proximate causes of major cardiovascular events such as myocardial infarction and stroke [26].

Central to this process is lipid accumulation. The long-standing “lipid hypothesis” posits that hypercholesterolemia, particularly elevated levels of ApoB-containing lipoproteins such as LDL and cholesterol-rich remnants, is a fundamental trigger of atherogenesis [27]. These particles penetrate the dysfunctional endothelium and become retained in the intima, where they undergo oxidative and enzymatic modification. This promotes the recruitment of circulating monocytes, which differentiate into macrophages and internalize modified lipoproteins via scavenger receptors [28]. The uncontrolled uptake of these lipids exceeds cellular anti-inflammatory defenses, generating intracellular danger signals that activate endothelial cells and tissue-resident macrophages via pattern recognition receptors such as Toll-like receptors (TLRs), thereby initiating a sustained inflammatory cascade. In turn, inflammation itself also alters lipid metabolism. Indeed, inflammatory cytokines can impair cholesterol efflux from foam cells and induce hepatic dyslipidemia, creating a vicious circle of lipid-driven inflammation [28].

Increasing evidence suggests that innate immunity plays a central role in atherosclerotic lesion formation [8,28]. Supporting this notion, the accumulation of cholesterol crystals promotes activation of the nucleotide-binding oligomerization domain (NOD)-, leucine-rich repeat (LRR)- and pyrin domain-containing protein 3 (NLRP3) containing inflammasome within macrophages and triggers the release of interleukin (IL)-1β and IL-18, potent cytokines that amplify inflammation and recruit additional leukocytes [29]. Activated macrophages also secrete tumor necrosis factor α (TNF-α), IL-6, and chemokines, perpetuating local inflammation. The metabolic state of macrophages determines their inflammatory character: excess free fatty acids and oxidized LDL drive a pro-inflammatory M1 phenotype, which relies on glycolytic metabolism, secretes IL-1β and IL-6, and fosters foam cell formation [28,30]. In contrast, macrophages alternatively activated by IL-4, IL-13, or IL-10 adopt an M2 phenotype, engaging in oxidative metabolism, enhancing cholesterol efflux, secreting anti-inflammatory mediators, and stabilizing plaques via collagen production [30]. Advanced lesions often show predominance of inflammatory macrophages and a large necrotic core (dead foam cells and lipid debris), reflecting chronic unresolved inflammation. This innate immune imbalance, too many inflammatory foam cells, insufficient resolving macrophages, is a central driver of plaque expansion and instability.

Taken together, mechanistic insights from the lipid hypothesis, combined with the detection of immune cell infiltrates and pro-inflammatory mediators in human plaques, have firmly established atherosclerosis as a chronic inflammatory disease. Clinical observations underscore this interplay: although potent LDL-lowering therapies, including statins and PCSK9 inhibitors, reduce cardiovascular events, many patients still experience myocardial infarction despite controlled cholesterol, highlighting the contribution of residual arterial inflammation. In this context, growing clinical data suggest that lipid-driven and immune-mediated mechanisms are not mutually exclusive, but rather closely interconnected. Pivotal clinical trials such as Canakinumab Anti-inflammatory Thrombosis Outcomes Study (CANTOS) [31] have shown that targeting inflammation through IL-1β inhibition reduces the risk of myocardial infarction and stroke independently of lipid levels [32]. Similarly, the Colchicine Cardiovascular Outcomes Trial (COLCOT) demonstrated that low-dose colchicine, an anti-inflammatory agent, also lowers the incidence of recurrent ischemic events [33]. These findings emphasize that dampening immune activation provides additive benefits beyond cholesterol lowering. However, broad immunosuppression carries risks, including heightened susceptibility to infections, underscoring the need for precision immunotherapy. Collectively, these observations have given rise to an “immune-metabolic paradigm” in which both lipid and immune/inflammatory pathways are strategically targeted to optimally manage atherosclerotic disease.

These findings underscore that targeting inflammation can provide additive clinical benefits beyond those achieved by cholesterol-lowering therapy alone. Modern research, therefore, frames atherosclerosis as a syndrome of maladaptive immune-metabolic interplay, where lipids act as drivers of immune activation, and immune responses exacerbate lipid dysregulation. Altogether, this evolving understanding has led to the emergence of an immune-metabolic paradigm, in which atherosclerosis is managed through the strategic integration of both lipid-modifying and immune-targeting therapies.

## 3. Cholesterol-Lowering Drugs Also Anti-Inflammatory

Among the primary cholesterol-lowering therapies are statins and, more recently, PCSK9 inhibitors. Both drug classes were originally developed with the core objective of lowering LDL-C, a key contributor to ASCVD. Statins inhibit HMG-CoA reductase, reducing hepatic cholesterol synthesis and upregulating LDL receptors, thereby effectively lowering plasma LDL-C concentrations [17,19]. PCSK9 inhibitors are monoclonal antibodies that prevent the degradation of LDL receptors, further enhancing LDL-C clearance [20,21] and providing cardiovascular benefits, as shown in clinical trials and mechanistic studies [22,23]. However, despite achieving target LDL-C levels, a significant proportion of patients continue to experience cardiovascular events. This residual risk has been increasingly attributed to persistent vascular inflammation, referred to as residual inflammatory risk, reflected by elevated inflammatory markers such as high-sensitivity C-reactive protein (hs-CRP) [34]. Addressing this risk requires improved patient phenotyping to distinguish lipid-mediated from inflammation-mediated residual risk, identification of biomarkers that reflect plaque inflammation more accurately than systemic hs-CRP, and development of combination therapies that integrate lipid lowering with targeted anti-inflammatory approaches [31,35].

While the cholesterol-lowering effects of these agents are well established and extensively reviewed elsewhere [36,37], this perspective focuses on their pleiotropic anti-inflammatory properties (Table 2). Growing evidence indicates that modulation of vascular inflammation substantially contributes to cardiovascular protection, providing the rationale for exploring these additional mechanisms in detail.

### 3.1. Cholesterol Synthesis Inhibitors

Statins not only inhibit cholesterol synthesis via HMG-CoA reductase but also enhance LDL uptake by increasing LDLR expression [38], and modulate inflammatory signaling through inhibition of the mevalonate pathway, which reduces downstream isoprenoid formation necessary for prenylation of small GTPases (e.g., Rho) [39], thereby affecting nuclear factor-kappa B (NF-κB) and other inflammatory pathways. Experimental and clinical studies show that statins reduce inflammasome activation, lower inflammatory cytokines, and decrease expression of inflammasome components such as NLRP3 and caspase-1, as well as TLRs and other innate immune sensors [40,41,42]. Mechanistically, statins have been demonstrated to reduce IL-1β release from peripheral blood mononuclear cells stimulated with cholesterol crystals and to inhibit NLRP3 activation in monocyte/macrophage models [41,43]. Clinically, statin therapy lowers circulating inflammatory markers such as hs-CRP, and intensive statin treatment is associated with a reduction in cardiovascular event rates, partly independent of LDL-C reduction, supporting pleiotropic anti-inflammatory benefits [44,45] (Table 2). In addition, recent evidence indicates that statins modulate novel glycosylation-related inflammatory biomarkers [46]. Statin therapy has been shown to modestly reduce circulating levels of glycoprotein acetylation markers such as GlycA, which reflect systemic inflammation [24], suggesting a potential influence of statins on inflammatory pathways involving protein glycosylation related to atherosclerosis.

Bempedoic acid, another cholesterol synthesis inhibitor that blocks the adenosine triphosphate (ATP)-citrate lyase (ACLY) upstream of HMG-CoA reductase, has also shown promising anti-inflammatory effects. Each of the series of the ACL-Inhibiting Regimen (CLEAR) Outcomes trial showed significant reductions in hs-CRP levels [47,48,49,50]. Thus, although further studies are warranted to clarify the molecular mechanisms underlying the Bempedoic acid anti-inflammatory effects, the CLEAR trial highlights the anti-inflammatory properties similar to statins.

### 3.2. PCSK9 Inhibitors

Monoclonal antibodies targeting PCSK9 (e.g., evolocumab, alirocumab) dramatically lower LDL-C by binding circulating PCSK9, preventing it from inducing lysosomal degradation of LDLR. This preservation of LDLR enhances their recycling to the hepatocyte surface, thereby increasing hepatic clearance of LDL-C from the circulation. Emerging evidence suggests that PCSK9 may also directly promote vascular inflammation [16,20]. PCSK9 inhibition has been linked with favorable changes in human atherosclerotic plaques, including reduced expression of NLRP3, IL-1β, and other inflammatory markers, along with improved plaque morphology in observational and mechanistic studies [16,20]. Large randomized trials confirmed significant reductions in LDL-C and major cardiovascular events, effects primarily attributed to lipid lowering, while systemic inflammatory biomarker changes (e.g., hs-CRP) were modest or inconsistent [16,20]. Nevertheless, beyond classical lipid and inflammatory effects, recent studies suggest that PCSK9 inhibitors also influence glycosylation-associated inflammatory biomarkers. According to recent data [51], PCSK9 blockade modulates circulating glycoprotein markers such as GlycA and GlycB, which are linked to systemic inflammation and plaque activity. This suggests that PCSK9 inhibitors may contribute to cardiovascular protection through mechanisms involving modulation of glycosylated acute-phase proteins, adding complexity to their immune-metabolic effects.

### 3.3. Ezetimibe

Ezetimibe reduces intestinal cholesterol absorption and, when combined with statins, provides incremental LDL-C lowering. The Improved Reduction in Outcomes: Vytorin Efficacy International Trial (IMPROVE-IT) demonstrated additional cardiovascular event reduction with ezetimibe added to statin therapy after acute coronary syndrome [52]. The anti-inflammatory effects of ezetimibe are less clear: some studies report reductions in hs-CRP and other inflammatory markers when ezetimibe is added to statins, whereas others suggest minimal independent anti-inflammatory activity of ezetimibe alone [53,54]. Overall, ezetimibe’s anti-inflammatory impact appears largely mediated via enhanced LDL lowering and synergy with statin pleiotropic effects.

To date, studies have not explicitly reported that ezetimibe reduces circulating GlycA or GlycB levels. While some experimental models, particularly in the context of diabetes, have shown that ezetimibe may influence glycation or glycoxidation processes, such as reducing levels of advanced glycation end-products (AGEs) like carboxymethyl-lysine (CML), these effects have primarily been observed in animal models [55]. In contrast, a study in patients with diabetes and hypercholesterolemia showed that ezetimibe did not significantly alter glycoalbumin levels compared to placebo [56]. Collectively, this suggests that although ezetimibe may have some impact on glycation-related pathways, there is currently no robust evidence that it modifies glycosylated acute-phase protein markers in human studies.

### 3.4. Direct Anti-Inflammatory Therapy: Canakinumab (CANTOS)

The CANTOS trial directly tested the hypothesis that targeting inflammation, independent of lipid lowering, can reduce cardiovascular events. In patients with prior myocardial infarction and elevated hs-CRP, canakinumab, a monoclonal antibody inhibiting IL-1β, administered every three months, significantly reduced major adverse cardiovascular events compared to placebo without materially altering LDL-C levels [31]. This pivotal study provided proof-of-concept for anti-inflammatory interventions in atherosclerosis, though its clinical use is limited by cost, infection risks, and patient selection challenges.

**Table 2:** Summary of interventions, study type, inflammatory effects, and cardiovascular impact.

Intervention	Study Type	Effect on Inflammation	Effect on Cardiovascular Risk/Plaque	Reference
Experimental studies				
Simvastatin	Experimental (human PBMCs stimulated with cholesterol crystals)	↓ IL-1β release and inflammasome activation in PBMCs; ↓ NLRP3 signaling	Indirect: statins reduce hs-CRP and clinical events in many trials (pleiotropic effects)	[41]
Atorvastatin	Experimental (human cell line)	↓ NLRP3 inflammasome via suppression of TLR4/MyD88/NF-kB pathway; ↓ IL-1β cleaved caspase-1	na (preclinical study)	[40]
Ezetimibe	Experimental (in vivo, diabetic rats)	↓ AGE	na (preclinical study)	[55]
Clinical studies				
Rosuvastatin	Two nested CVD case–control studies: JUPITER and TNT trials (primary and secondary prevention)	IgG N-glycans profile associated with incident CVD: an agalactosylated glycan related to increased risk of CVD, several digalactosylated and sialylated IgG glycans related to decreased risk	IgG glycan score was positively associated with future CVD	[46]
Anti-inflammatory therapies in ASCVD (systematic review)	Systematic review (clinical studies)	↓ hs-CRP, ↓ IL-6 (summarized effects across several trials)	Mixed evidence for ↓ MACE depending on therapy	[44]
Rosuvastatin (JUPITER trial)	Clinical interventional trial (RCT)	↓ hs-CRP	↓ MACE in patients with normal LDL-C	[45]
	IgG glycosylation traits, GlycA	Carotid and femoral plaques	Yes	[11]
Canakinumab (anti-IL-1β) (CANTOS)	Clinical trial (RCT)	↓ IL-1β and ↓ hs-CRP	↓ MACE independent of LDL-C	[31]
Ezetimibe + statin (IMPROVE-IT study)	Clinical trial (randomized controlled trial)	↓ LDL and hs-CRP (mixed data across studies)	↓ CV events when ezetimibe added to statins post-ACS	[52,53]
PCSK9 inhibitors (alirocumab; observational, GlycA/B/F markers)	Observational (pre-/post in high CV risk subjects)	↓ GlycA ~12%, GlycF, GlycB; ↓ ApoC-III and TG; minimal/ no change in hs-CRP	Major ↓ LDL-C; indirect implications for CV risk reduction	[51]
PCSK9 inhibitors (evolocumab/alirocumab)	Clinical observational (human carotid plaques)	↓ plaque pro-inflammatory proteins (NLRP3, IL-1β, TNFα)	Plaque stabilization features, ↓ MACE	[16]
Dietary interventions (Mediterranean diet, omega-3 PUFA)	Clinical trial (RCT, cohort)	↓ inflammatory markers (IL-6, CRP)	↓ CV risk	[57,58]

When shown, a downward arrow (↓) represents downregulation. Abbreviations: ACS, acute coronary syndrome; ApoC-III, apolipoprotein C-III; AGE, advanced glycation end-products; ASCVD, atherosclerotic cardiovascular disease; CANTOS, Canakinumab Anti-inflammatory Thrombosis Outcomes Study (NCT01327846); CV, cardiovascular; CVD, cardiovascular disease; hs-CRP, high sensitive C reactive protein; IgG, immunoglobulin G; IL-1β, interleukin 1β; IL-6, interleukin 6; IMPROVE-IT, Improved Reduction in Outcomes: Vytorin Efficacy International Trial (NCT00202878); JUPITER, Justification for the Use of Statins in Prevention: an Intervention Trial Evaluating Rosuvastatin (NCT00239681); LDL-C, low-density lipoprotein cholesterol; MACE, major adverse cardiovascular events; MyD88, myeloid differentiation primary response 88; NF-κB, nuclear factor κB; NLRP3, Nucleotide-binding oligomerization domain (NOD)-, leucine-rich repeat (LRR)- and pyrin domain-containing protein 3; na, not assessed; PBMCs, peripheral blood mononuclear cells; PCSK9, Proprotein convertase subtilisin/kexin 9; RCT, Randomized clinical trials; TG, triglycerides; TLR4, toll-like receptor 4; TNT trial, Treating to New Targets; (NCT00327691).

## 4. Conclusions and Perspectives

Cholesterol metabolism and immune activation are tightly linked in the pathogenesis of atherosclerosis. Cholesterol accumulation, defective efflux, and lipid modification generate metabolic “danger” signals that trigger innate immune sensors, prime inflammasomes, and promote a sustained pro-inflammatory milieu that drives atherosclerotic plaque progression. Cholesterol-lowering therapies, particularly statins and PCSK9 inhibitors, not only reduce lipid burden but also exert anti-inflammatory effects through diverse mechanisms. Moreover, other dedicated anti-inflammatory interventions, such as IL-1β blockade in CANTOS, have demonstrated that reducing inflammation can lower cardiovascular event rates independently of LDL-C levels.

In this context, composite biomarkers of glycosylated acute-phase proteins, such as GlycA and GlycB, have emerged as informative indicators of systemic inflammation and vascular risk. These biomarkers integrate inflammatory signals across multiple circulating glycoproteins and have been consistently associated with atherosclerosis burden and adverse cardiometabolic outcomes. Although the evidence remains limited, some studies suggest that cholesterol-lowering therapies, including statins and PCSK9 inhibitors, may modestly influence these glycosylation-related biomarkers, suggesting that part of their cardiovascular benefits could involve attenuation of immune-metabolic dysregulation in addition to LDL-C lowering.

The pro-inflammatory environment in adverse cardiometabolic conditions, including obesity, diabetes, and metabolic syndrome, can be further amplified, thereby intensifying immune cell activation and systemic and local inflammation, which significantly increases the risk of CVD progression and related complications in affected subjects [59]. Therefore, a combined immune-metabolic therapeutic approach, along with improved inflammatory biomarkers to guide treatment, appears to be the most promising path forward for effective cardiovascular risk reduction, mainly in such cardiometabolic conditions.

## 5. Future Directions

Significant advances have been made over recent decades in our understanding of immune cell dysfunction in CVD. This progress has increased attention on the immune-metabolic networks that regulate cellular function. Metabolic stressors are known to substantially alter cellular metabolism, providing valuable new insights into the complexities and commonalities of immune cell metabolism involved in chronic inflammatory diseases, including atherosclerosis. In this context, current metabolic drugs, originally developed as cholesterol-lowering agents, have also demonstrated effectiveness in treating other inflammatory conditions. For example, PCSK9 inhibition during the severe inflammatory stage of severe acute respiratory syndrome coronavirus 2 (SARS-CoV-2) infection reduced the primary endpoint of death or need for intubation, as well as IL-6 levels [60]. Similarly, patients treated with statins prior to hospitalization had lower mortality from SARS-CoV-2 infection [61]. Looking ahead, a deeper understanding of the pleiotropic, cholesterol-independent anti-inflammatory effects of conventional lipid-lowering drugs, such as PCSK9 inhibitors and statins, may reveal novel mechanisms of action and therapeutic potential to improve cardiovascular outcomes beyond their lipid-lowering properties.

Key gaps include understanding which patients benefit most from direct anti-inflammatory therapies, determining the optimal timing (primary vs. secondary prevention), identifying safe long-term immunomodulation strategies, and developing biomarkers reflecting plaque biology. Based on current evidence, several avenues warrant further investigation to optimize cardiovascular protection through immune-metabolic modulation. Integrated therapeutic strategies combining cholesterol-lowering agents with targeted inflammasome modulators may synergistically attenuate plaque inflammation and reduce cardiovascular events. Translational research to map immune-metabolic states of human plaque immune cells (single-cell metabolic profiling, spatial transcriptomics, metabolomics) will be important to identify novel targets that modulate lipid handling and inflammation simultaneously [62,63]. The development of plasma, including glycated protein patterns or imaging biomarkers that accurately reflect immune-metabolic states, may enable patient-specific, precision therapy. Translational studies employing preclinical models to test combinatorial interventions, such as statins in combination with NLRP3 inhibitors, are needed to validate both efficacy and the underlying mechanisms. Finally, lifestyle and metabolic interventions, including diet, exercise, and pharmacological modulators of metabolism, may modulate immune responses within plaques [57,58] (Table 2), offering promising non-pharmacological adjuncts to conventional therapy. Collectively, these approaches underscore the potential of an integrated immune-metabolic paradigm for personalized cardiovascular care.

## Figures and Tables

**Table 1:** Studies evaluating glycated proteins and glycosylation traits as biomarkers of subclinical atherosclerosis.

Study	Biomarker(s)	Atherosclerosis Outcome	Independence from LDL	Reference
Quantification of glycoproteins by ^1^H-NMR in T1D	GlycA, GlycB, GlycF (H/W ratios)	Carotid plaque presence and number	Yes	[10]
IgG glycosylation profile and subclinical atherosclerosis	IgG glycosylation traits, GlycA	Carotid and femoral plaques	Yes	[11]
GlycA and PAD/Carotid Plaque (MESA)	GlycA (^1^H-NMR)	Carotid plaques, ABI	No/attenuated after LDL adjustment	[13]
Glycated ApoB as a surrogate marker	Serum glycated ApoB	Carotid intima-media thickness, plaque presence	No/lipid-mediated	[12]

^1^H-NMR, proton nuclear magnetic resonance; T1D, Type 1 diabetes; IgG, immunoglobulin G; PAD, peripheral artery disease; MESA, Multi-Ethnic Study of Atherosclerosis; ABI, ankle–brachial index; LDL, low-density lipoprotein cholesterol; ApoB, apolipoprotein B.

## Data Availability

When writing the manuscript, the authors did not have access to any special sets of data. As such, the authors cannot provide any special access to datasets that readers might request.

## Abbreviations

The following abbreviations are used in this manuscript:

ACLY	Adenosine triphosphate (ATP)-citrate lyase
AGE	Advanced glycation end-products
ApoB	Apolipoprotein B
ApoC-III	Apolipoprotein CIII
ASCVD	Atherosclerotic cardiovascular diseases
CANTOS	Canakinumab Anti-Inflammatory Thrombosis Outcomes Study
CLEAR	ACL-Inhibiting Regimen
CML	Carboxymethyl-lysine
COLCOT	Colchicine Cardiovascular Outcomes Trial
CVD	Cardiovascular disease
GlycA	Glycoprotein A
GlycB	Glycoprotein B
hs-CRP	High-sensitivity C-reactive protein
HMG-CoA	3-hydroxy-3-methylglutaryl-coenzyme A
^1^H-NMR	Proton nuclear magnetic resonance
IgG	Immunoglobulin
IL	Interleukin
IMPROVE-IT	Improved Reduction in Outcomes: Vytorin Efficacy International Trial
JUPITER	Justification for the Use of Statins in Prevention: An Intervention Trial Evaluating Rosuvastatin
LDL-C	Low-density lipoprotein cholesterol
LDLR	Low-density lipoprotein receptor
MESA	Multi-Ethnic Study of Atherosclerosis
NF-κB	Nuclear factor-kappa B
NLRP3	Nucleotide-binding oligomerization domain (NOD)-, leucine-rich repeat (LRR)-, and pyrin domain-containing protein 3
PBMCs	Peripheral blood mononuclear cells
PCSK9	Proprotein convertase subtilisin/kexin 9
RCT	Randomized clinical trials
ROS	Reactive oxygen species
TLR	Toll-like receptor
TNF-α	Tumor necrosis factor α
TNT	Treating to New Targets
T1D	Type 1 diabetes
